# Measurement of Dabigatran Concentration Using Finger Prick Dried Blood Spot Sample Collection

**DOI:** 10.3389/fphar.2021.679431

**Published:** 2021-05-26

**Authors:** Shin-Yi Lin, Yu-Fong Peng, Chih-Fen Huang, Ching-Hua Kuo, Sung-Chun Tang, Jiann-Shing Jeng

**Affiliations:** ^1^ Department of Pharmacy, National Taiwan University Hospital, Taipei, Taiwan; ^2^ School of Pharmacy, College of Medicine, National Taiwan University, Taipei, Taiwan; ^3^ Stroke Center and Department of Neurology, National Taiwan University Hospital, Taipei, Taiwan

**Keywords:** dabigatran, finger prick test, therapeutic drug monitoring, non-vitamin K antagonist oral anticoagulants, drug concentration

## Abstract

**Background and Purpose:** Real-world laboratory monitoring of dabigatran activity is challenging. The purpose of the present study was to demonstrate the feasibility and accuracy of finger prick sampling with dried blood spot (fpDBS) cards in measuring the dabigatran concentration.

**Material and Methods:** Patients >20 years of age with atrial fibrillation and receiving dabigatran therapy for more than 7 days were included in the study. Peak and trough dabigatran concentrations were collected by simultaneous finger prick and venous puncture. The dabigatran concentration was measured by ultra-high performance liquid chromatography with tandem mass spectrometry. Our previously developed post-column infused internal standard (PCI-IS) method was applied to estimate the blood spot volume on fpDBS and to calibrate the drug concentration. Deming regression was used to analyze the correlation between dabigatran concentration on fpDBS cards and in plasma samples, followed by Bland–Altman analysis to compare the bias between two sampling techniques.

**Results:** A total of 33 patients were enrolled and contributed 66 plasma and 55 fpDBS dabigatran samples. The average patient age was 74.6 ± 7.9 years, mean creatinine clearance 58.1 ± 18.3 mL/min, and CHA_2_DS_2_-VASc score 3.5 ± 1.6 points. The dabigatran concentration ranged from 41.8–1421.7 ng/mL. The plasma and DBS dabigatran concentrations correlated well (*r* = 0.98), and the conversion factor for fpDBS to plasma dabigatran concentration was 1.28. The Bland–Altman analysis showed that 94.5% of the fpDBS-predicted concentration fell within 20% of bias.

**Conclusions:** The study showed that fpDBS measurement of dabigatran concentration is reliable and can be applied in clinical scenarios.

## Introduction

Dabigatran is a direct thrombin inhibitor effective in preventing stroke and systemic embolism in atrial fibrillation (AF) patients, with a reduced risk of intracranial hemorrhage (ICH) compared to warfarin (Connolly et al., 2009). The pharmacokinetic and pharmacodynamic relationship of dabigatran is predictable; therefore, routine laboratory testing is not necessary (Steffel et al., 2018). However, the relationship between concentration and clinical outcomes of dabigatran was reported in the Randomized Evaluation of Long-Term Anticoagulation Therapy (RE-LY) trial. Both ischemic stroke and major bleeding outcomes correlated with dabigatran concentration (Reilly et al., 2014). In real-world data, dabigatran concentration increased in specific populations, including the elderly (age ≥75 years), patients with renal impairment (creatinine clearance [CrCL] ≤50 mL/min), patients who are thin (weight ≤60 kg), and patients with more co-morbid disease, reflected by a CHA_2_DS_2_-VASc score >3 points and HAS-BLED score ≥3 points (January et al., 2019). More importantly, the dabigatran concentration could also be influenced by behavior. Low dabigatran concentration was associated with poor drug adherence (January et al., 2019). Therefore, in emergent circumstances and among specific populations, measuring the dabigatran concentration may be essential and beneficial.

The finger prick dried blood spot (fpDBS) sampling technique collects a small amount of blood on filter paper via finger prick, followed by a drying process.The procedure is simple, minimally invasive, and facilitates sample transportation and storage (Li and Tse, 2010). The fpDBS sampling allows patients to perform therapeutic drug monitoring at home, reduces the cost of blood sample storage, and enhances the stability of labile compounds (Enderle et al., 2016; Nijenhuis et al., 2016; Wilhelm et al., 2014; Wang et al., 2015; McDade, 2014; ter Heine et al., 2011). We previously developed and validated a DBS-based assay to quantify NOAC by ultra-high performance liquid chromatography with tandem mass spectrometry (UHPLC-MS/MS) (Foerster et al., 2018; Jhang et al., 2020). The post-column infused internal standard (PCI-IS) method was used to estimate spot volume and adjust the matrix effect to improve the accuracy of quantification (Jhang et al., 2020). Whole spot extraction could be used with the PCI-IS analytical approach, minimizing blood spreading error due to hematocrit (HCT) variation. When using PCI-IS to measure drug concentration in fpDBSs, no specially designed device to control spot volume is required for blood sampling, which makes the proposed method practical for various clinical settings.

Although the fpDBS sampling approach has many advantages, especially when considering precision medicine, only one study has investigated the correlation of dabigatran concentration between fpDBS and plasma samples (Foerster et al., 2018). That study only enrolled six patients, and HCT information was not provided. These limitations make it difficult to evaluate the clinical utility of the fpDBS approach.

To elucidate the utility of fpDBS sampling for determining dabigatran concentrations in real-world practice, the current study enrolled 33 patients who contributed 66 plasma and 55 fpDBS samples. Detailed clinical characteristics, including HCT levels, were collected, and the samples were measured using the PCI-IS method. The concentration correlation was studied and predicted accuracy of plasma concentration calculated to demonstrate the feasibility and accuracy of fpDBS cards in measuring the dabigatran concentration.

## Material and Methods

### Participants and Setting

This was a prospective observational study conducted in a tertiary hospital in Taiwan. Patients aged >20 years who were on dabigatran for more than 7 days were included in the study. Patients. who failed to provide simultaneous fpDBS and plasma samples or refused to provide written informed consent were excluded. The study protocol was approved by the International Ethics Committee of National Taiwan University Hospital.

### Blood Sample Collection

Blood samples were collected 1–4 hours after dabigatran administration (peak) or immediately before the next dabigatran dose (trough). A total of 5 mL of blood was collected in K_2_EDTA tubes (BD Vacutainer^®^). The fpDBS sample was collected via finger prick with a lancet. Whole blood was dropped onto Whatman 903 Protein Saver Cards (Maidstone, United Kingdom) to create blood spots. The fpDBS card was allowed to dry at room temperature for at least 2 hours, and then was stored at −20°C.

Plasma samples were obtained by centrifugation at 3,000 rcf for 15 min and stored at −80°C. A 100 μL aliquot of human plasma was used to measure the dabigatran concentration.

### Sample Preparation

Primary stock solutions of dabigatran and [^13^C_6_]-dabigatran, each at a concentration of 100 μg/mL, were prepared separately in methanol and stored at −20°C. Aliquots of the stock solutions were spiked into blank plasma samples and blank blood samples to prepare working standards for the calibration curve.

The blood spots on the fpDBS cards were cut into a clean Eppendorf tube using a manual puncher. A total of 300 μL of water containing 0.1% (v/v) formic acid and 10 ng/mL [^13^C_6_]-dabigatran was added and the samples extracted using a Geno/Grinder 2010 (SPEX^®^ Sample Prep, Metuchen, NJ) for 2 min at 1,1000 rpm. A 700 µL aliquot of acetonitrile was then added to the formic acid extract and extracted using the Geno/Grinder 2010 for another 3 min. After centrifugation at 18,000 rcf for 5 min, 800 μL of supernatant was placed into another Eppendorf tube and evaporated using an EYELA CVE-200D centrifugal evaporator (Tokyo Rikakikai Co, Tokyo, JP).The dried samples were stored at −20°C before reconstitution. To process the plasma samples, a total of 800 μL MeOH containing 2 ng/mL [^13^C_6_]-dabigatran was added to the 100 μL plasma sample and homogenized by the Geno/Grider 2010at 1,000 rpm for 2 min. After deproteinization, the sample was centrifuged at 15,000 rpm for 5 min. The 800 μL of supernatant was collected and then dried in an EYELA CVE-200D centrifugal evaporator overnight. The dried samples were stored at −20°C before reconstitution. MeOH (200 μL) was used for sample reconstitution, followed by filtration for UHPLC-MS/MS analysis (0.22-μm PP membrane filters; RC-4, Sartorius, Göttingen, Germany).

### Dabigatran Concentration

The dabigatran concentration was measured by UHPLC-MS/MS. Detailed analytical conditions were published previously (Jhang et al., 2020). All LC analyses were performed using an Agilent 1290 UHPLC system (Agilent Technologies, Waldbronn, Germany). A Kinetex reversed-phase core-shell C18 column (2.1 × 50 mm, 2.6c µm, 100 Å, Phenomenex, Torrance, CA, United States) was used as the separation column. The mobile phase consisted of 0.1% formic acid, 10 mM ammonium acetate in 40% ACN (solvent A) and 0.1% formic acid, and 10 mM ammonium acetate in ACN/IPA (A/I; 1:9) (solvent B) in gradient mode. The flow rate was 0.35 mL/min and the injection volume was 3 μL.

The MS analysis was performed using an Agilent 6460 triple quadrupole system (Agilent Technologies, Waldbronn, Germany). The positive electrospray ionization mode was used with the following parameters: dry gas temperature 350°C, dry gas flow rate 10 L/min, nebulizer pressure 45 psi, sheath gas temperature 350°C, sheath gas flow rate 11 L/min, nozzle voltage of 500 V, and capillary voltage of 3500 V. The sample analysis was performed in multiple reaction monitoring mode (MRM) and the transition pairs were 472.2–289 (quantifier) and 472.2–144 (qualifier) for dabigatran and 478.2–295.1 for [^13^C_6_]-dabigatran.

The method validation was performed according to FDA guidance (U.S. Department of Health and Human Services Food and Drug Administration Center for Drug Evaluation and Research (CDER) Center for Veterinary Medicine (CVM), 2018). Detailed validation procedures were reported in our previous article (Jhang et al., 2020). In brief, the precision and accuracy were investigated at low, medium, and high concentrations. The stability study test was performed at low and high concentrations. For inter-day precision, three different samples per concentration were analyzed at three separate days. The results for intra-day and inter-day precision were determined as relative standard deviation (RSD%). The accuracy was reported as the percent recovery (measured concentration relative to the target concentration). For every ten real sample injections during the analytical procedure, one QC injection was inserted to ensure the quality and repeatability of the analytical system. Validation results showed that the accuracy was 85–115% and the precision was less than the RSD of 15%. The RSD of the quality control samples was within 2%. Stability study revealed dabigatran was stable under −20°C for 30 days (Jhang et al., 2020).

### Using PCI-IS for Blood Volume Estimation on fpDBS Cards

PCI-IS was used to estimate the blood volume of fpDBSs. We selected 1000 ng/mL LysoPE in methanol (17:1) and infused it at a flow rate of 0.1 mL/min. The PCI-IS chromatogram at the first ion suppression zone (from 0.345 to 0.385 minutes) was used to represent the degree of ion suppression caused by the salts in the blood sample. A blood sample from a healthy volunteer was used to generate a calibration curve from 10–40 μL of blood on the fpDBS cards against the reciprocal of the response of the PCI-IS chromatogram at the first ion suppression zone with a total seven points (*n* = 3 for each points).

The method validation in this study was performed according to FDA guidance for bioanalytical method validation (U.S. Department of Health and Human Services Food and Drug Administration Center for Drug Evaluation and Research (CDER) Center for Veterinary Medicine (CVM), 2018). Blood sample from a healthy volunteer was used to generate a calibration curve from 10 to 35 µL of blood on DBS cards. To evaluate the estimation accuracy and precision, blood samples from seven other individuals were spotted on DBS cards with volumes of 10, 15, 20, 25, 30, and 35 μL (*n* = 3 for each volume). The estimate volume relative to the target volume was used to calculate the accuracy. The precision was determined as RSD%. Validation of the blood volume estimation showed that the accuracy was 85–115% and the precision was <15% RSD. The estimated blood volume was then used to correct the concentration of dabigatran.

### HCT Measurement

We adapted clinical biochemistry test results for the HCT levels of blood samples using the Sysmex XE-5000 automated hematology system (Sysmex Canada Inc., Mississauga, Ontario, Canada). If the data were not available, we recorded the HCT level within 30 days of fpDBS collection.

### Statistical Analysis

Descriptive analyses were applied to obtain the mean and standard deviation or median and interquartile range (IQR). To evaluate the correlation between dabigatran concentrations in plasma and fpDBS samples, Deming regression analysis was applied, followed by Bland−Altman analysis to compare the predicted and actual plasma dabigatran concentration. All data analyses were performed in Microsoft Excel (Microsoft, Redmond, WA, United States) and IBM SPSS Statistics (version 26.0; IBM Corp, Armonk, NY, United States).

## Results

**FIGURE 1:**
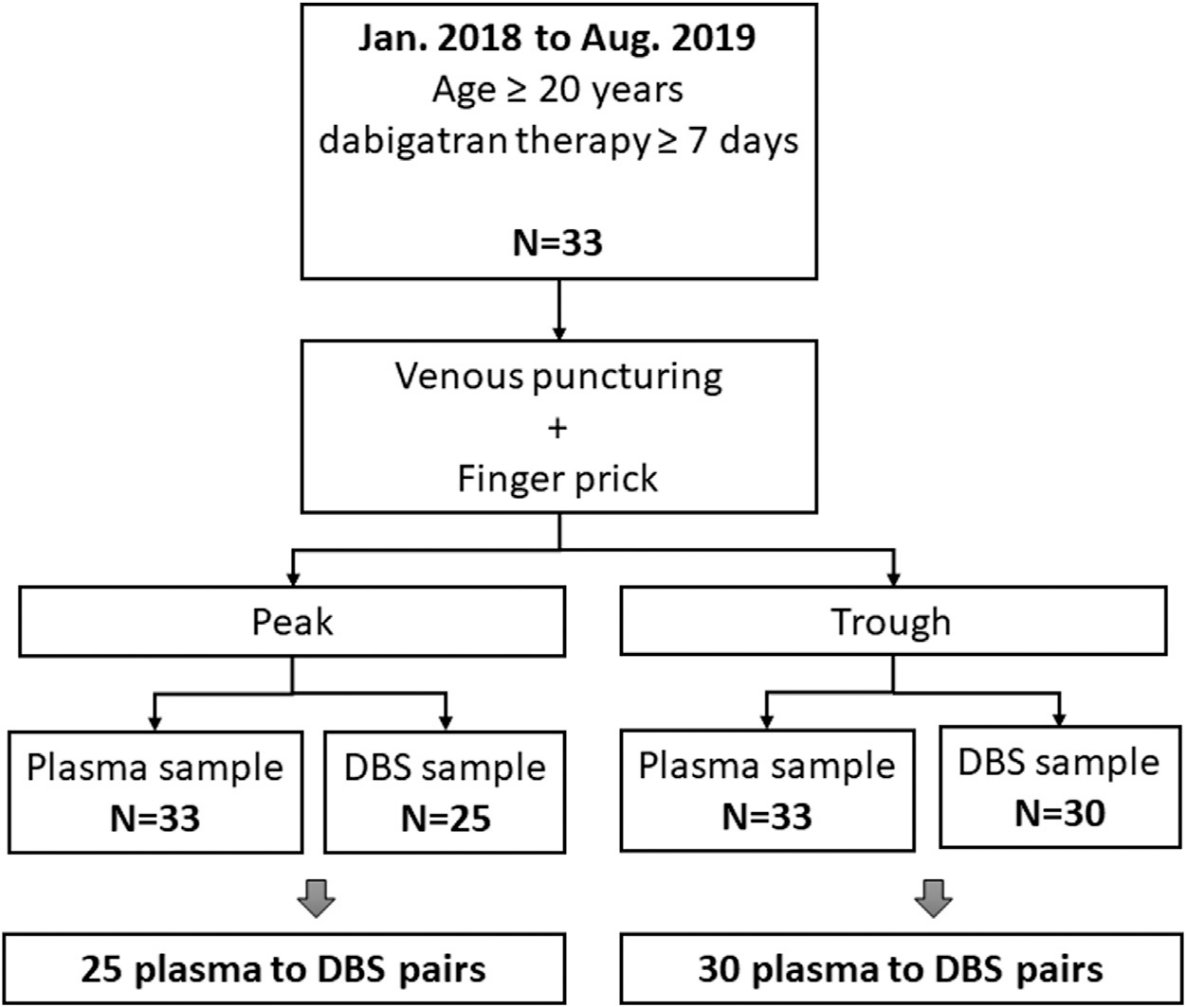
The process of study enrollment.

From January 2018 to August 2019, a total of 33 participants were enrolled in the study. Detailed baseline characteristics are given in Table 1. Participant enrollment is depicted in Figure 1.

**TABLE 1:** Demographic characteristics for the study participants.

Characteristic	
Age (year)	74.5 ± 7.9
Male	24 (72.7%)
Weight (kg)	71.0 ± 12.7
Serum creatinine (mg/dL)	1.0 ± 0.2
Creatinine clearance (mL/min)	58.1 ± 18.4
Hematocrit (%), *n* = 25	42.9 ± 3.7
CHA_2_DS_2_VASc^a^	3.5 ± 1.6
HAS-BLED^b^	2.1 ± 0.8
Co-morbidities	
Ischemic stroke or transient ischemic attack	10 (15.2%)
Congestive heart failure	4 (6.1%)
Hypertension	23 (34.8%)
Diabetes	8 (12.1%)
MI or PAOD	5 (7.6%)
Bleeding history	5 (7.6%)
Concurrent medications^c^	
Aspirin	1 (1.5%)
Amiodarone	4 (6.1%)
Verapamil	1 (1.5%)
Non-steroidal anti-inflammatory drugs	3 (4.5%)
Dabigatran regimen	
110 mg twice daily	22 (66.7%)
150 mg twice daily	11 (33.3%)
Dabigatran level (ng/mL)	
Trough (plasma, with DBS pair), *n* = 30	292.8 193.5
Dose normalized trough (plasma)	2.4 ± 1.7
Trough (DBS), *n* = 30	322.9 ± 214.6
Dose normalized trough (DBS)	2.0 ± 1.5
Sample volume (µL)	10.6 ± 2.7
Peak (plasma, with DBS pair), *n* = 25	555.8 ± 320.6
Dose normalized peak (plasma)	4.5 ± 2.7
Peak (DBS), *n* = 25	426.6 ± 221.1
Dose normalized peak (DBS)	3.7 ± 2.1i
Sample volume (µL)	10.2 ± 2.7

MI, myocardial infarction; PAOD, peripheral arterial vascular disease; TIA, transient ischemic attack.

a CHA_2_DS_2_VASc score: To evaluate the risk for ischemic stroke among patients with atrial fibrillation. Higher score indicates higher risk of ischemic stroke. For CHA_2_DS_2_VASc score, the additional risk factors including assigning one point to age 65–74 years, female sex, or vascular disease and two points to age ≥75 years.

b HASBLED score: To evaluate the risk for bleeding. Higher score indicates higher risk. One point is assigned to hypertension, abnormal liver function, abnormal renal function, stroke history, bleeding history, labile international normalized ratio (INR) during warfarin therapy, age over 65 years, antiplatelet agent, non-steroidal anti-inflammatory drug or ethanol use. The item labile INR was not calculated in the present study.

c Concurrent medications: medications which has interaction with dabigatran were listed. None of our patients concomitantly used clopidogrel, quinidine, azole antifungal agents, protease inhibitors (P-glycoprotein inhibitors), and rifampin, enzyme inducing antiepileptic drugs such as phenytoin and phenobarbital (P-glycoprotein inducers).

### Correlation Between fpDBS and Plasma Dabigatran Concentrations

All participants contributed 33 peak and 33 trough plasma samples, but eight peak fpDBS samples and three trough fpDBS samples were not available. The cause of unavailable fpDBS samples was failure to obtain sufficient blood spot size and participant preference of not to repeat the test. In total, there were 55 pairs of fpDBS and plasma samples from 30 participants. The median plasma dabigatran peak and trough concentrations were 501.0 (IQR 376.0–761.6 ng/mL) and 262.2 (IQR 147.9–378.3 ng/mL). For fpDBS, the dabigatran peak and trough concentrations were 409.5 (IQR 278.7–555.75 ng/mL) and 181.3 (IQR 123.5–291.7 ng/mL). The fpDBS volume was 10.4 ± 2.7 μL. Overall, the plasma concentration ranged from 41.8 to 1,421.7 ng/mL, and the fpDBS concentration ranged from 35.0 to 926.4 ng/mL.

**FIGURE 2:**
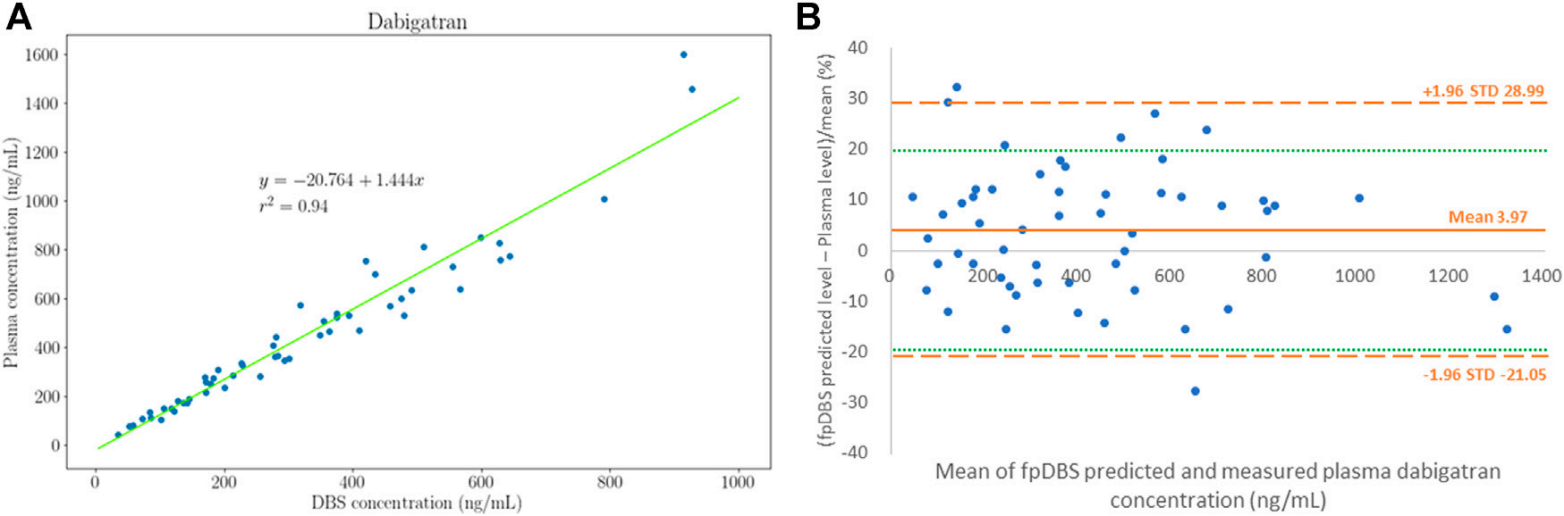
Distribution of plasma and finger prick dried blood sampling (fpDBS) dabigatran concentration. **(A)** The correlation of paired fpDBS and plasma dabigatran concentration, and **(B)** Bland–Altman plot for dabigatran concentrations that depict the % differences between estimated and measured plasma dabigatran concentrations.

The plasma dabigatran concentration correlated well with the fpDBS dabigatran concentration. Pearson’s correlation coefficient was 0.98. The Deming regression between plasma and fpDBS dabigatran concentration is depicted in Figure 2A. The conversion factor calculated from the average ratio of plasma to fpDBS concentration was 1.28 ± 0.15 (mean and standard deviation), indicating that the plasma dabigatran concentration was higher than the concentration in the fpDBS sample. The result of the Bland−Altman analysis is given in Figure 2B. The fpDBS predicted concentration was estimated by multiplying measured fpDBS concentration by the conversion factor, 1.28. With the exception of three blood samples with fpDBS predicted bias >20% (maximum 28.5%), 94.5% of fpDBS predicted concentrations fell within 20% of bias.

### Impact of HCT on fpDBS Predicted Dabigatran Concentration

**FIGURE 3:**
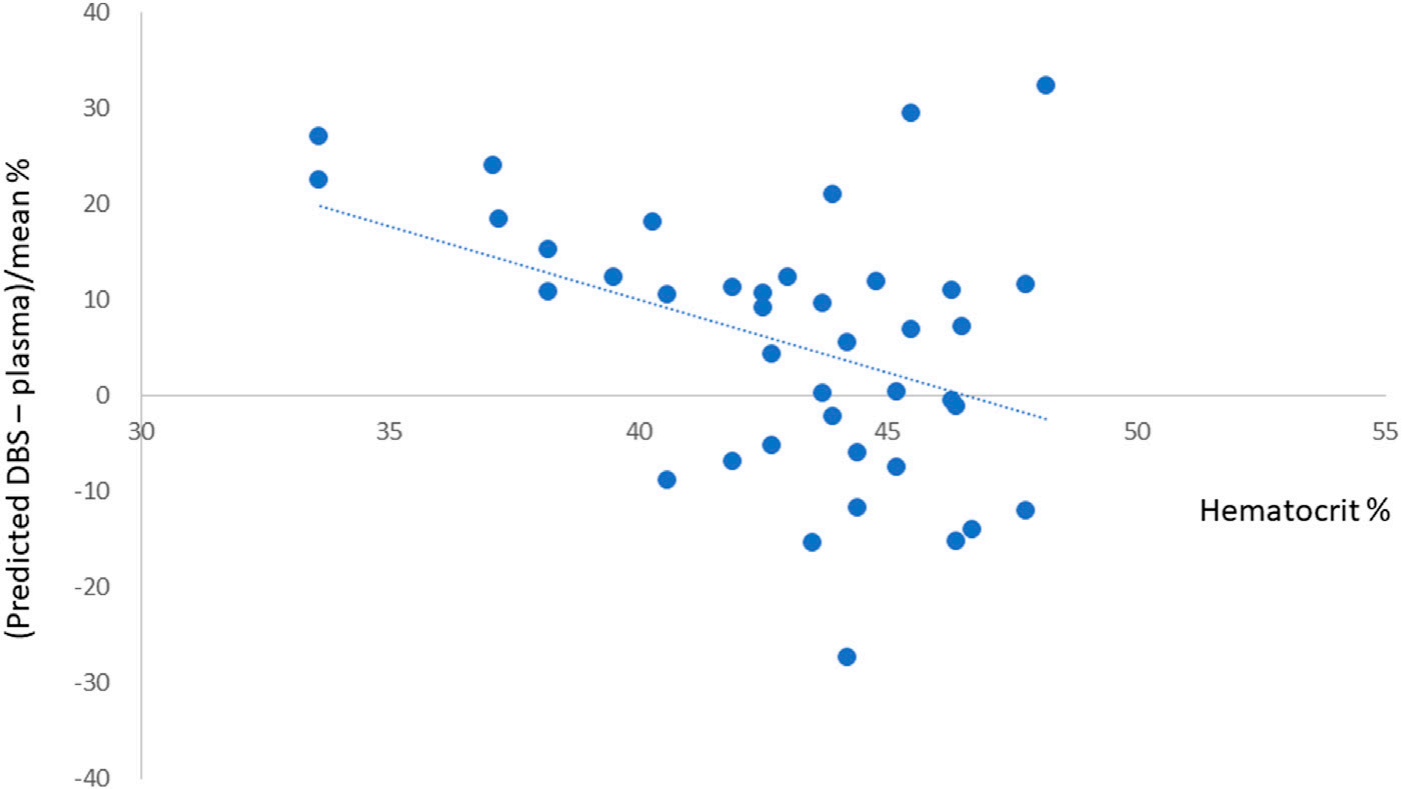
The impact of hematocrit level to bias of DBS predicted dabigatran concentration.

The HCT level was available for 40 samples. The mean HCT level was 43.0 ± 3.6% (range 33.6–48.2%). The correlation between bias, calculated from the percent difference between the predicted and measured plasma dabigatran concentration and HCT level is depicted in Figure 3. Positive bias was observed at low HCT and negative bias observed at high HCT. The linear relationship can be displayed as bias% = −1.49(HCT)+67.11. The *R* ^2^ was 0.17. The bias was generally <20% except for three samples with HCT of 33.6, 45.5%, and 48.2%.

## Discussion

Our investigation determined the correlation between the fpDBS card and plasma of dabigatran concentrations, finding good correlation between the two sampling techniques with a conversion factor of 1.28 for fpDBS to plasma. Few studies have tested the reliability of using fpDBS sampling to monitor dabigatran concentration in clinical practice. Foerster et al. analyzed the relationship between fpDBS and plasma dabigatran concentrations and reported a conversion factor of 1.51 (Foerster et al., 2018). In our previous investigation, the conversion factor was 1.73 (Jhang et al., 2020). There could be several reasons for the inconsistency in conversion factors across different studies. First, in our previous investigation, we used venous blood and a pipette to ensure consistent spot volume. However, in the present study, we collected dried blood samples by finger prick. In addition to different blood sources, collecting capillary blood adds variation in test results, such as errors from calibration for blood volume. Nevertheless, we observed a high correlation between the plasma and fpDBS concentrations with more than 90% of fpDBS predicted concentration falling within 20% of bias, indicating that the PCI-IS method still had good calibrating performance for blood volume.

Second, consistent with our previous investigation, positive bias was observed with low HCT levels, whereas negative bias was noted with increased HCT levels (Jhang et al., 2020). This could be explained by the distribution of dabigatran mainly in the plasma (Schmid et al., 2019). The contribution of HCT to the recovery difference has been tested before and the bias found to be <10%. Therefore, we proposed that adjusting the dabigatran concentration according to HCT was unnecessary (Jhang et al., 2020). In the present study, we examined the HCT level in three patients with >20% difference in the Bland−Altman analysis. Although the distribution of HCT in these three samples was at the lower and higher end of all participants, the errors could not be explained by the HCT effect on plasma-blood distribution. Therefore, we assumed the bias may result from analytical error and correcting for HCT is not necessary in fpDBS analysis.

Inter-individual differences existed in different study groups. Clinical trials and real-world data all showed that the dabigatran concentration changes across different demographic characteristics (Connolly et al., 2009; January et al., 2019; Lin et al., 2019). The dabigatran concentration range in our study was broad (41–1,400 ng/mL) compared to Foerster et al. investigation in which all were <400 ng/mL (Foerster et al., 2018). Further, compared to the data reported in clinical trials, which was 28–215 ng/mL for trough concentration and 52–383 ng/mL for peak concentration, around 65% of plasma-fpDBS pair had concentration being higher-than-expected range dabigatran concentrations (Reilly et al., 2014; Steffel et al., 2021). The cause for increased dabigatran concentration can be multifocal, including ethnicity, and patient characteristics, such as higher proportion elderly, renal impairments, and higher CHA_2_DS_2_-VASc score. Most importantly, selection bias may exist due to our small participant number. The distribution of dabigatran concentration observed in present study may not fully reflect the results for Asian population. Nevertheless, our data displayed good correlation between fpDBS and plasma concentration. The fpDBS sampling is accurate, convenient, and applicable in clinical practice for measuring dabigatran concentration not only within a wide concentration range (i.e., 41–1,400 ng/mL), but with a tiny sample size (10 μL).An increased dabigatran concentration was associated with increased risk of bleeding according to the RE-LY study (Reilly et al., 2014). Real-world data also reported major bleeding events during dabigatran therapy (Chen et al., 2020). Measuring the dabigatran concentration is essential and beneficial in specific populations (Douxfils et al., 2018).

We acknowledge the following limitations of our study. First, though the UHPLC-MS/MS is highly sensitive, we did not enroll participant with very low dabigatran concentration. Whether the correlation between fpDBS and plasma dabigatran concentration can be extrapolated patient with concentration lower than 40 ng/mL remains uncertain. Nevertheless, as mentioned before, the recovery, precision and accuracy for low dabigatran concentration (i.e., 10 ng/mL) has been tested in our previous study and the results were adequate (Jhang et al., 2020). Second, we measured unconjugated rather total dabigatran concentration, and the reported concentration in this study did not include glucuronides. According to literature reports, the proportion for its contribution to total dabigatran concentration ranges from less than 10–35% (Saffian et al., 2015; Stangier et al., 2010). Further studies with sample hydrolysis treatment could consider the contribution of glucuronides in fpDBS. Third, despite our investigation developed good correlation between fpDBS and plasma dabigatran concentration, the UHPLC-MS/MS method is not routinely available in most laboratories. The turnaround time for dabigatran concentration measurement is more than 1 week and make the test not feasible in urgent circumstance. Nevertheless, therapeutic monitoring for dabigatran users is still helpful to identify patients with change in dabigatran exposure and ensure the quality of anticoagulant therapy. Forth, we did not compare UHPLC-MS/MS method to other tests such as HEMOCLOT thrombin inhibitor assays, diluted thrombin time, and ecarin clotting time for dabigatran concentration measurement because all of the latter were not available in our laboratories. Fifth, the number of participants in this study was relatively small, and most of them were elderly. Whether the results appropriately reflect the dabigatran distribution in whole Asian population remain unclear. Larger scale investigations with a broader variety of participant characteristics are necessary. Last, sample collection in this study was performed at a hospital by well-trained healthcare practitioners. Thus, special considerations for home sampling were not discussed. A future study with home sampling fpDBSs is necessary to demonstrate fpDBS sampling as a feasible self-care approach for improving the safety and efficacy of dabigatran treatment. In conclusion, our study provided real-world data on using fpDBS sampling to measure dabigatran concentration, showing that the method is valuable in clinical practice.

## Data Availability

The raw data supporting the conclusion of this article will be made available by the authors, without undue reservation.
